# Machine Learning-Aided Optimization of In Vitro Tetraploid Induction in Cannabis

**DOI:** 10.3390/ijms26041746

**Published:** 2025-02-18

**Authors:** Marzieh Jafari, Nathan Paul, Mohsen Hesami, Andrew Maxwell Phineas Jones

**Affiliations:** Department of Plant Agriculture, University of Guelph, Guelph, ON N1G 2W1, Canada

**Keywords:** classification, leaf-related morphological traits, optimization algorithm, oryzalin, plant tissue culture, polyploidy

## Abstract

Polyploidy, characterized by an increase in the number of whole sets of chromosomes in an organism, offers a promising avenue for cannabis improvement. Polyploid cannabis plants often exhibit altered morphological, physiological, and biochemical characteristics with a number of potential benefits compared to their diploid counterparts. The optimization of polyploidy induction, such as the level of antimitotic agents and exposure duration, is essential for successful polyploidization to maximize survival and tetraploid rates while minimizing the number of chimeric mixoploids. In this study, three classification-based machine learning algorithms—probabilistic neural network (PNN), support vector classification (SVC), and k-nearest neighbors (KNNs)—were used to model ploidy levels based on oryzalin concentration and exposure time. The results indicated that PNN outperformed both KNNs and SVC. Subsequently, PNN was combined with a genetic algorithm (GA) to optimize oryzalin concentration and exposure time to maximize tetraploid induction rates. The PNN-GA results predicted that the optimal conditions were a concentration of 32.98 µM of oryzalin for 17.92 h. A validation study testing these conditions confirmed the accuracy of the PNN-GA model, resulting in 93.75% tetraploid induction, with the remaining 6.25% identified as mixoploids. Additionally, the evaluation of morphological traits showed that tetraploid plants were more vigorous and had larger leaf sizes compared to diploid or mixoploid plants in vitro.

## 1. Introduction

Polyploidy, defined as the condition of having more than two complete sets of chromosomes per cell nucleus, plays a crucial role in plant evolution and diversification [1]. While diploid organisms possess two sets of chromosomes, polyploid organisms can have multiple sets, such as triploids with three, tetraploids with four, and octoploids with eight [2,3]. This phenomenon is notably prevalent in agriculture, with up to 40% of cultivated crops being polyploids, presumably due to the phenotypic implications such as larger leaves, flowers, and fruits [4]. For example, modern bananas are triploid [5], potatoes are generally tetraploid [6], wheat is hexaploid [7], and cultivated strawberries are octoploid [8], illustrating the diverse application and significance of polyploidy in crop development and improvement.

In cannabis, polyploidy induction has garnered significant attention for its potential to enhance cannabinoid production and overall plant vigor [9,10,11]. Cannabis is typically a diploid species; however, instances of naturally occurring triploids and tetraploids have been reported [12,13,14]. Despite the promise, research on artificial polyploid induction in cannabis is still in its early stages, with only a few studies conducted to date and conflicting results likely resulting from genotypic variability [15,16,17,18]. This highlights critical gaps in the literature that underscore the need for further investigation to fully understand and harness the benefits of polyploidy in this economically and medically important crop.

Generally, polyploidy results in several significant biological and agricultural advantages, including larger cell sizes that can translate into increased dimensions of stems, roots, leaves, flowers, and fruits [1,19,20,21]. It also alters secondary metabolite profiles, which can affect plant chemistry and interactions with their environment in various ways [22]. Additionally, polyploidy enhances tolerance to various abiotic stresses in some species, such as drought and salinity tolerance, contributing to plant resilience [23,24,25]. Further, multiple copies of each gene can result in more and different allelic combinations to generate a wide range of phenotypic diversity, which is beneficial for targeted selection in breeding programs [26,27]. This also increases the potential for heterosis, or hybrid vigor, potentially leading to more robust and productive hybrid plants [28,29]. Of particular relevance to cannabis, it can also facilitate the production of seedless plants, especially at odd levels of ploidy, which is valuable to facilitate the production of seedless cannabis flowers in environments where pollen may be present [5].

To generate artificial polyploids, antimitotic agents are used to disrupt the natural cell cycle [30,31]. During mitosis, chromosomes duplicate during interphase, align along the cell’s equator in metaphase, and are separated by spindle fibers during anaphase, resulting in the division of the cell into two daughter cells [32]. The application of antimitotic agents, such as oryzalin, can alter this process by inhibiting the formation of functional spindles [22]. As a result, proper chromosome segregation is disrupted, and following cell division, tetraploid cells are produced. This ability to induce polyploidy through antimitotic agents offers a valuable tool for plant breeding and genetic studies [22]. However, polyploidy induction faces several challenges, primarily due to the complex nature of meristems that consist of numerous cells in various stages of the cell cycle, and not all cells respond uniformly to antimitotic agents [33]. This can lead to the production of mixoploids, a chimeric form of polyploidy where cells within the same organism exhibit different levels of ploidy, such as both diploid and tetraploid cells [34]. This heterogeneity poses a significant hurdle, as mixoploids can revert to the diploid state, making it difficult to obtain stable polyploid lines [33]. Achieving consistent and uniform polyploidy is crucial to producing stable tetraploid clonal lines and for reliable use in plant breeding programs [35].

The concentration of antimitotic agents (e.g., oryzalin and colchicine) and the duration of exposure are critical factors influencing the success of polyploidy induction and can impact the rate of mixoploid production [22,36]. To optimize these conditions, it is essential to choose the appropriate oryzalin concentration and exposure time to maximize tetraploid induction with minimal mixoploid formation, which necessitates testing various combinations of oryzalin concentrations and exposure times [35]. Traditionally, this is done using a factorial experimental design, but this requires large numbers of treatments and is often logistically challenging. For instance, testing 10 concentrations at 10 different exposure times results in 100 treatments, making the process tedious, time-consuming, and costly. Moreover, there is a concern that the optimal combination might be among the levels excluded from the treatments. To address these challenges, the application of machine learning (ML) presents a promising avenue for optimizing these conditions more efficiently [37].

Machine learning, a subfield of artificial intelligence, involves the creation of predictive models using provided data and algorithms [38,39]. It encompasses supervised, unsupervised, and reinforcement learning methods [40]. In tissue culture, supervised ML is commonly employed to develop in vitro culture protocols [41]. This approach utilizes datasets containing features such as hormone concentrations and labels, including polyploidy level and regeneration rate. Labels in supervised machine learning are further categorized into quantitative parameters like shoot regeneration rate and qualitative parameters such as ploidy level. Depending on the type of label, supervised machine learning is further classified into regression for quantitative data and classification for qualitative data [37]. Previous studies have demonstrated the applicability and reliability of regression-based ML algorithms in predicting and optimizing various in vitro culture systems such as seed germination [42], direct organogenesis [43], shoot proliferation [44], callogenesis [45], androgenesis [46], somatic embryogenesis [47], rooting [48], and gene transformation [49].

In relation to polyploid induction, classification-based ML is suitable for predicting whether a micro-propagated plant is tetraploid, diploid, or mixoploid. This process involves training a model using labeled data, where each instance is associated with a class label indicating its ploidy level. The model learns patterns and features from the input data to make predictions about the class membership of new, unseen instances [41]. Classification algorithms, such as support vector machines, K-nearest neighbors, and neural networks, are commonly used in this context to achieve accurate and reliable classification results [37]. This application of ML offers a valuable tool for identifying and selecting tetraploids in polyploidy engineering studies.

The main objective of this study was the optimization of conditions for tetraploid induction in cannabis. Therefore, the current study was performed (i) to evaluate the effect of different concentrations of oryzalin at various exposure times on in vitro tetraploid induction, (ii) to evaluate and compare the applicability and reliability of different classification-based ML algorithms for modeling and predicting the level of ploidy, (iii) to optimize oryzalin concentrations and exposure durations to maximize tetraploid induction using an optimization algorithm, and finally (iv) to evaluate and compare different leaf-related morphological traits in diploid and tetraploid plants.

## 2. Results

### 2.1. Effects of Different Concentrations of Oryzalin and Different Exposure Times on Tetraploid Induction

**Figure 1:**
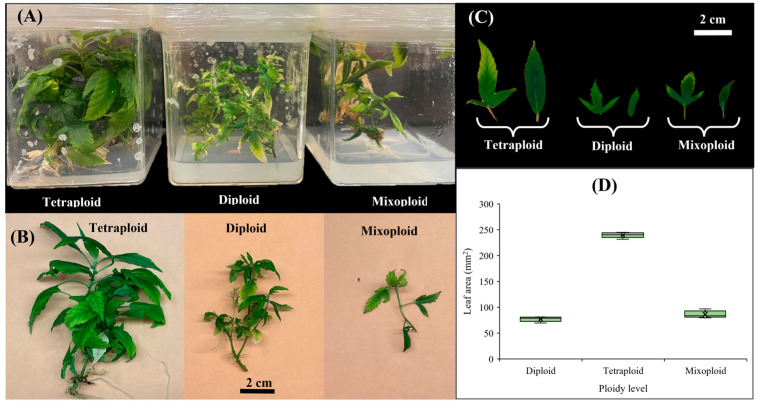
In vitro polyploidy induction in cannabis: (**A**) in vitro-grown plantlets with different ploidy levels, (**B**) plantlets with different ploidy levels after 6 weeks, (**C**) leaves of tetraploid, diploid, and mixoploid plantlets, and (**D**) leaf area (mm^2^) of tetraploid, diploid, and mixoploid plantlets.

Different treatments resulted in varying responses, including diploid, mixoploid, and tetraploid (Figure 1A).

**Figure 2:**
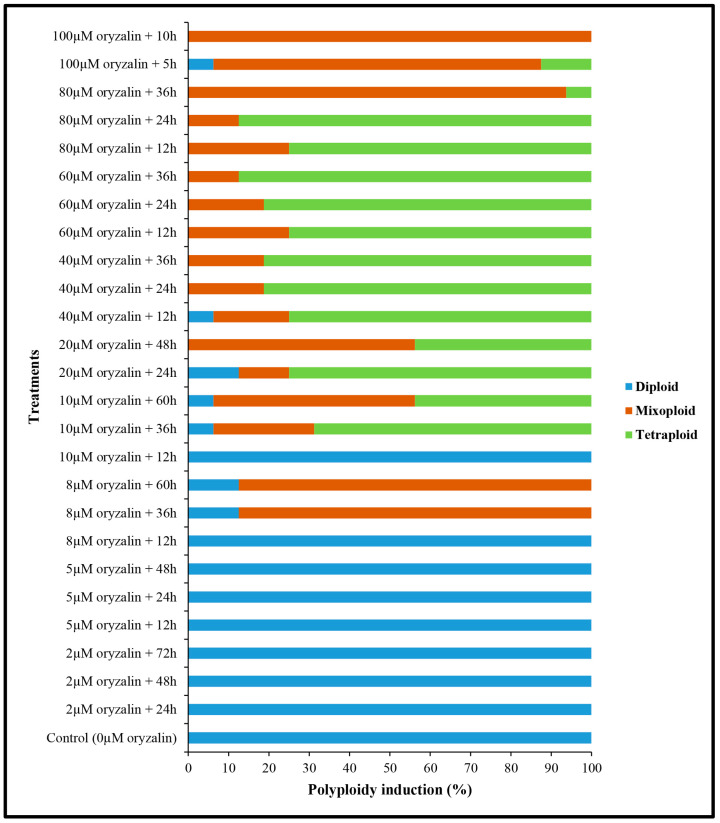
Effect of different oryzalin concentrations at various exposure times on in vitro polyploidy induction in cannabis.

For instance, 0, 2, and 5 µM of oryzalin for different exposure times, as well as 10 µM of oryzalin for 12 h produced only diploid plants, while 100 µM of oryzalin for 10 h resulted in the induction of mixoploid plants (Figure 2). The highest rate (87.5%) of tetraploid induction was observed in 60 µM of oryzalin + 36 h and 80 µM of oryzalin + 24 h, followed by 40 µM of oryzalin + 24 h, 40 µM of oryzalin + 36 h, and 60 µM of oryzalin + 24 h at 81.25% tetraploid induction rate (Figure 2).

### 2.2. Leaf-Related Morphological Traits in Diploid, Mixoploid, and Tetraploid Plants

**Figure 3:**
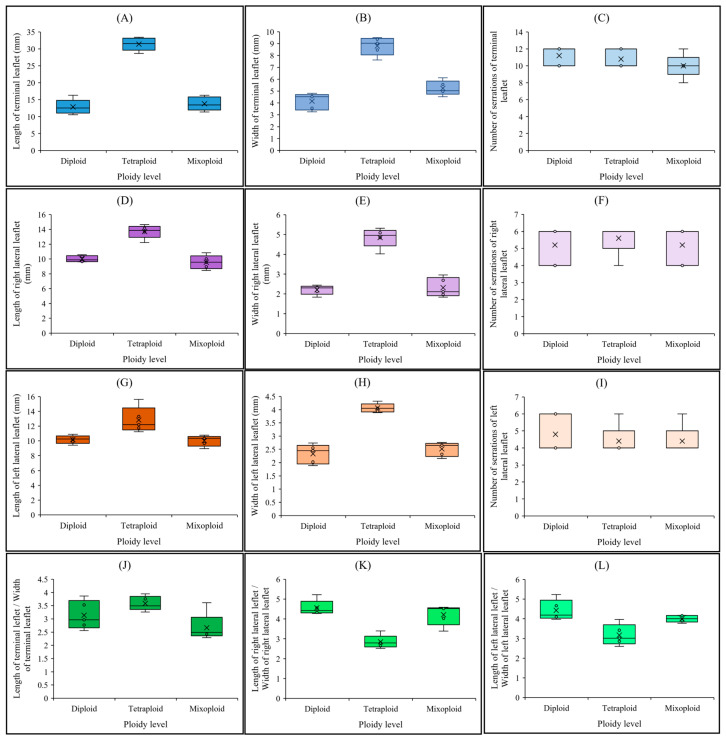
Leaf-related morphological traits in cannabis with different ploidy levels, including diploid, tetraploid, and mixoploid; (**A**) length of the terminal leaflet, (**B**) width of the terminal leaflet, (**C**) number of serrations of the terminal leaflet, (**D**) length of the right lateral leaflet, (**E**) width of the right lateral leaflet, (**F**) number of serrations of the right lateral leaflet, (**G**) length of the left lateral leaflet, (**H**) width of the left lateral leaflet, (**I**) number of serrations of the left lateral leaflet, (**J**) length of the terminal leaflet/width of terminal leaflet ratio, (**K**) length of the right lateral leaflet/width of right lateral leaflet ratio, (**L**) length of the left lateral leaflet/width of left lateral leaflet ratio.

As shown in Figure 1B, there were significant differences between tetraploids and diploids. Tetraploids produce larger and more vigorous plants compared to diploids (Figure 1B). The leaves of tetraploid plants were nearly triple the size of those of diploid or mixoploid plants (Figure 1C). For instance, the leaf area of tetraploid plants (239.77 ± 5.24 mm^2^) was approximately three times larger than that of diploid (77.18 ± 4.90 mm^2^) or mixoploid (86.04 ± 6.88 mm^2^) plants (Figure 1D). The length of the terminal leaflet (Figure 3A) in tetraploid plants (31.42 ± 1.92 mm) was approximately twice that of diploid (12.86 ± 2.19 mm) or mixoploid (13.81 ± 2.02 mm) plants. A similar pattern was observed for the width of the terminal leaflet (Figure 3B), as well as the width of both the right (Figure 3E) and left (Figure 3H) lateral leaflets. Although the length of the right (Figure 3D) and left (Figure 3G) lateral leaflets in tetraploids was higher than in both diploid and mixoploid plants, there were negligible differences in the number of serrations in both the terminal (Figure 3C) and lateral leaflets (Figure 3F,I) across all ploidy levels. In addition, tetraploid plants showed no significant differences in the leaflet length-to-width ratios for the terminal (Figure 3J), right lateral (Figure 3K), and left lateral (Figure 3L) leaflets compared to diploid plants. This indicates that the lengths and widths of both terminal and lateral leaflets in diploid and tetraploid plants grew synchronously.

**Figure 4:**
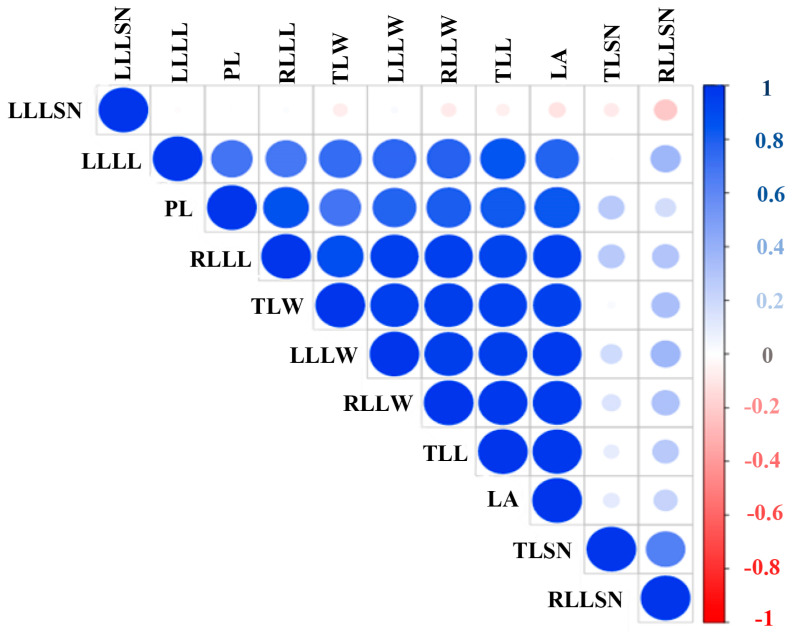
Correlation between cannabis leaf-related morphological traits and ploidy levels. LA: leaf area; LLLL: left lateral leaflet length; LLLSN: left lateral leaflet serration number; LLLW: left lateral leaflet width; PL: ploidy level; RLLL: right lateral leaflet length; RLLSN: right lateral leaflet serration number; RLLW: right lateral leaflet width; TLL: terminal leaflet length; TLSN: terminal leaflet serration number; TLW: terminal leaflet width.

According to the correlation analysis (Figure 4), the ploidy level exhibited significant positive correlations with leaf area, the length of the terminal leaflet, the length of the right lateral leaflet, the length of the left lateral leaflet, the width of the terminal leaflet, the width of the right lateral leaflet, and the width of the left lateral leaflet. However, there were no significant correlations between ploidy levels and leaf marginal patterning (i.e., the number of serrations in the terminal leaflet and both the right and left lateral leaflets) (Figure 4).

### 2.3. Evaluation and Comparison of the Developed Machine Learning Models

The performances of the PNN, SVC, and KNN models were evaluated using different performance criteria. The results of the training and testing phases of each model are detailed in Table 1. The PNN model demonstrated high performance during both the training and testing phases, achieving an accuracy of 96.4912% in training and 86.6667% in testing. The precision, recall, and F_1_-score values were consistently high, indicating a robust model with balanced performance metrics (Table 1). The KNN model achieved an accuracy of 92.9825% during training and 80% during testing. While the training phase exhibited strong performance, the testing phase indicated a drop in accuracy and consistency across precision, recall, and F_1_-score values, suggesting potential overfitting (Table 1). The SVC model showed an accuracy of 80.7018% in the training phase and maintained a similar accuracy of 80% during testing. However, the precision and recall values varied significantly between the training and testing phases, resulting in a lower F_1_-score value during testing, which indicates that the model might struggle with generalization (Table 1). Among the three models, the PNN exhibited the highest overall performance, with superior accuracy, precision, recall, and F_1_-score values in both the training and testing phases (Table 1).

### 2.4. Optimization Process and Experimental Confirmation of Predicted-Optimized Conditions

**Figure 5:**
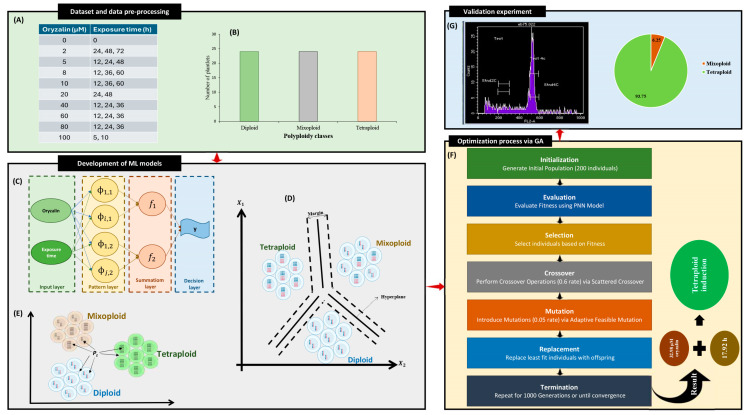
Schematic representation of the data-driven approach for modeling and optimizing in vitro tetraploid induction in cannabis; (**A**) different treatments for generating the dataset, (**B**) the distribution of the plantlets in each ploidy level, (**C**) probabilistic neural network, (**D**) support vector classification, (**E**) k-nearest neighbors, (**F**) genetic algorithm, and (**G**) validation experiment.

Since the PNN model proved to be the most effective, the developed PNN model was linked to GA to optimize the oryzalin concentration and exposure duration to maximize tetraploid induction. The optimization process using GA indicated that a concentration of 32.98 µM of oryzalin for approximately 18 h would result in optimal tetraploid induction (Figure 5F). To validate the predictions of the PNN-GA hybrid model, a laboratory experiment was conducted. The validation experiment confirmed the model’s predictions, showing a 93.75% tetraploid induction rate and 6.25% mixoploidy (Figure 5G). As a result, the developed PNN-GA model demonstrated precise predictive and optimization capability for polyploid induction.

## 3. Discussion

Inducing polyploidy through antimitotic agents (e.g., colchicine and oryzalin) is a widely recognized method for chromosome duplication [22]. This technique is employed to generate polyploid strains, investigate the gigas effect, develop seedless fruits in triploid varieties, and potentially increase the production of secondary metabolites in medicinal plants [26,50]. In the specific case of cannabis, polyploid lines have been used to explore its potential to enhance cannabinoid production, overall plant vigor, and seedlessness [9,10,11,15,18]. The success rate of polyploid induction using oryzalin or colchicine is frequently low due to the mixoploid production and has been reported in both hemp varieties [16,17] and drug-type cannabis [18].

Indeed, the efficiency of polyploidy induction in cannabis, like many other crops, can be negatively affected by mixoploid induction [15,16,17,18]. It is thus necessary to develop methods that minimize mixoploidy to improve the success rate and utility of tetraploid induction. One promising strategy to address this bottleneck is the application of ML methods [37]. With the application of supervised ML techniques (i.e., regression- and classification-based supervised ML models) for detecting the complex patterns and relationships among the factors impacting in vitro culture systems, more efficient and productive in vitro culture systems can be established [41]. Various regression-based ML techniques have been employed to develop a predictive model in different cannabis in vitro culture systems, such as callogenesis [51], in vitro sterilization [52], in vitro shoot growth and development [53], and in vitro seed germination [54,55,56]. These studies highlight the robustness and reliability of using ML techniques in this context.

In the case of polyploidy induction, where the output is categorical, regression-based ML techniques are not well suited. However, classification-based ML algorithms provide a powerful alternative computational strategy for enhancing the efficiency of tetraploid induction [9]. Classification-based ML models identify ploidy levels (diploid, mixoploid, and tetraploid) in response to associated factors (level of oryzalin and exposure duration) by developing predictive models based on patterns within the dataset [41]. The developed models can then be employed to generate adaptive practices to maximize the induction of tetraploid plants while minimizing mixoploid production.

In this study, three different classification-based ML algorithms (SVC, KNNs, and PNN) were used to model and predict in vitro tetraploid induction based on associated factors (oryzalin concentration and exposure duration). The results showed that the PNN model outperformed both SVC and KNNs in terms of accuracy, error rate, precision, recall, and F_1_ score. One potential reason for the superior performance of PNN is its greater flexibility and adaptability compared to SVC and KNNs [57]. In contrast to KNNs, which depend on a set number of nearest neighbors for predictions [58,59,60], PNN can integrate a significantly larger number of data points into its decision-making process [61]. This capability enables PNN to identify more complex patterns and relationships within the dataset, leading to enhanced predictive accuracy [62]. As a probabilistic model, PNN assigns probabilities to each class based on the input data. This approach allows PNN to better account for noise and uncertainty within the dataset, potentially leading to more precise predictions [62].

Enhancing the efficiency of in vitro culture systems through the optimization of plant tissue culture procedures is pivotal, and GA, as a single-objective optimization algorithm, presents numerous advantages in this regard [63,64]. The efficiency of polyploid induction can vary widely depending on the plant species, type of explant, type and concentration of antimitotic agents, exposure duration, and desired goals [22]. GA offers adaptability and customization to address specific optimization challenges through the delineation of suitable genetic representations, fitness functions, and genetic operators [53]. GA can be regarded as a potent optimization algorithm for customizing the optimization process to meet the specific requirements of in vitro culture systems such as polyploid induction [37]. One particular benefit of employing GA for in vitro optimization is its capacity to effectively navigate through extensive search spaces [65]. In vitro culture systems such as tetraploid induction frequently encompass numerous variables, such as concentration of antimitotic agents, exposure time, and environmental factors. The interplay among these variables can give rise to intricate optimization challenges [22]. GA utilizes a method based on populations, where a collection of potential solutions, referred to as individuals, undergoes evolution over successive generations [66]. Through this population-based exploration, GA can simultaneously investigate a broad spectrum of parameter combinations, facilitating the identification of optimal solutions within a practical timeframe [37]. Another benefit of GA is its capability to address non-convexity and nonlinearity within the optimization landscape [53]. This characteristic renders GA especially well suited for discovering optimal solutions in non-convex and intricate optimization challenges linked with in vitro tetraploid induction.

Given that PNN proved to be the most accurate predictive model in our study, it was subsequently linked to a GA to optimize oryzalin concentration and exposure duration. This integration aimed to enhance the efficiency of tetraploid induction in cannabis. The results of the validation experiment illustrated that employing the predicted optimized condition (32.98 µM oryzalin for 18 h) through PNN-GA yielded a tetraploid induction rate of 93.75% in the validation experiment. Notably, this conversion rate is higher than reported in many previous studies. For instance, Parsons, Martin, James, Golenia, Boudko and Hepworth [18] reported a maximum tetraploid induction of 66.7% in drug-type cannabis utilizing 40 µM for 24 h. Kurtz, Brand and Lubell-Brand [16] demonstrated a maximum tetraploid induction of 64% in hemp cultivars employing 0.05% colchicine for 12 h. Further, it is worth highlighting that the conversion rate of 93.75% observed experimentally was greater than the treatments used in this study to develop the model. Hence, the validation experiment confirmed that the PNN-GA approach is a powerful and precise method for predicting in vitro polyploid induction in cannabis.

In addition to developing the in vitro tetraploid induction protocol, we investigated leaf-related morphological traits across diploid, mixoploid, and tetraploid plants. The principal attribute linked with polyploidy is the enlargement of plant organs, commonly referred to as the ‘gigas’ effect [67]. In this study, we found that not only was the plant more vigorous, but the leaf size was also larger in tetraploids than in diploids. In line with our results, Fernandes, et al. [68] reported that tetraploid cannabis plants exhibited significantly increased leaflet size compared to diploid plants, emphasizing the substantial impact of ploidy on plant morphology. Furthermore, our findings align with those of Parsons, Martin, James, Golenia, Boudko and Hepworth [18], who also reported that tetraploid plants have larger leaves than diploid plants. Specifically, the central leaflet on tetraploid leaves was significantly wider compared to that on diploid leaves [18]. These morphological changes were notable and have been documented across various plant species, such as *Citrus limonia* [69], *Malus × domestica* [67], *Anemone sylvestris* [70], *Brassica* species [71], and *Sorbus pohuashanensis* [72].

Our results showed that the leaf area of tetraploids was considerably higher than that of diploid plants. Although the length and width of terminal and lateral leaflets were significantly higher than that of diploids, the length and width of terminal and lateral leaflets in both diploid and tetraploid plants grew synchronously. Therefore, tetraploid plants exhibited no significant alteration in leaflet length-to-width ratios, aligning with findings from prior studies like those in birch [73]. It has been shown that the growth rate during the middle to late stages of leaf development dictates their eventual size [73]. Consequently, tetraploid leaves possessed a heightened growth rate compared to their diploid counterparts, which was an important reason for the large leaf size of tetraploid plants [74]. Studies in *Arabidopsis* showed that polyploidization would lead to larger cell areas [23]. Additionally, it was shown that there were no significant differences in cell proliferation ability among different ploidy levels, as assessed through the measurement of relative expression levels of cell division-related genes [23,74]. Hence, cell expansion stands out as a significant factor contributing to the increased size of tetraploid leaves. Indeed, the alteration primarily stems from the heightened DNA content, leading to an enlargement of the nucleus [75,76].

## 4. Materials and Methods

### 4.1. Plant Materials

**Figure 6:**
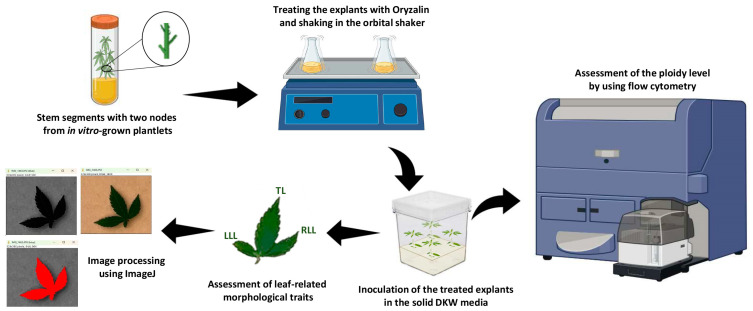
Schematic representation of the experimental methodology for in vitro tetraploid induction in cannabis, assessment of ploidy level, and measurement of leaf-related morphological traits. The scheme was created using BioRender.com.

Stem segments with two nodes from in vitro-grown drug-type cannabis (*Cannabis sativa* L. cv. Super Sherb) plantlets were selected as explant materials (Figure 6).

DKW [77] medium (D2470, PhytoTech Labs, Lenexa, KS, USA) supplemented with 30 g/L sucrose was used as the basal medium for this experiment. The pH of the medium was adjusted to 5.7 before autoclaving at 121 °C and 15 psi for 20 min. The explants were placed in a 500 mL Pyrex^®^ round media storage bottle (06-414-1C, Thermo Fisher Scientific, Waltham, MA, USA) containing 200 mL of liquid basal DKW medium supplemented with varying concentrations (0, 2, 5, 8, 10, 20, 40, 60, 80, and 100 µM) of oryzalin (O630, PhytoTech Labs, KS, USA). The inoculated media were then placed on an orbital shaker (160 rpm) and subjected to different exposure times (Figure 6). A total of 26 treatments were tested (Figure 5A), with each treatment having 4 replicates. Each replicate consisted of 4 explants. Following the oryzalin treatment, the explants were transferred into a Magenta GA7 vessel (50-255-176, Thermo Fisher Scientific, Waltham, MA, USA) containing solid DKW medium (containing 6 g/L agar) and kept at 27 °C, under an 18 h photoperiod, 50 µmol m^−2^ s^−1^ PPFD (photosynthetic photon flux density), comprised of 12.5% W (400–700 nm), 12.5% B (400–500 nm), and 75% R (600–700 nm) for 6 weeks in the growth room (Figure 6). After 6 weeks, polyploid levels in the treated explants were assessed using flow cytometry (Figure 6).

To assess ploidy level using flow cytometry, small leaf segments (approximately 1 cm^2^) were collected and kept on ice throughout preparation. The tissue was finely chopped with a sharp razor blade in a Petri plate containing 1000 µL of ice-cold LB01 buffer [78] composed of 15 mM Tris, 2 mM Na_2_EDTA, 0.5 mM spermine tetrahydrochloride, 80 mM KCl, 20 mM NaCl, 0.1% (*v*/*v*) Triton X-100, 25 µL of propidium iodide stock with a pH of 8.0. The resulting suspension was filtered through a 50 µm nylon mesh to remove large debris. The interval between sample preparation and flow cytometric analysis (FCM) was approximately 5 min. The stained nuclei were analyzed using a BD FACSCalibur flow cytometer (BD Biosciences, San Jose, CA, USA.) with an FL2 voltage setting of 486 V. Data for a minimum of 1000 nuclei per sample were captured within a maximum duration of 120 s. Relative DNA content was determined by measuring the fluorescence peak area using a 585/42 nm detector. Fluorescence peak means coefficients of variation and nuclei counts were recorded and analyzed using BD Accuri™ C6 Software 1.0.264.21. To ascertain ploidy levels, plant tissues with confirmed diploid status were used as a reference standard.

### 4.2. Leaf-Related Morphological Traits in Diploid, Mixoploid, and Tetraploid Plants

Leaf-related morphological traits of tetraploid, diploid, and mixoploid micro-propagated shoots were recorded using five replications, with each replicate consisting of eight leaves (Figure 6). Different leaf-related morphological traits, including length of lateral leaflets (left and right sides), length of terminal leaflets, width of lateral leaflets (left and right sides), width of terminal leaflets, number of serrations on terminal and lateral leaflets, and leaf area, were measured using ImageJ 1.53e based on the methodology of Hesami, et al. [79].

### 4.3. Dataset Description

After detecting outliers using principal component analysis (PCA), 72 data lines, each representing an experimental instance, were selected for further analyses. As shown in Figure 5B, the ploidy levels were divided into three classes (diploid, mixoploid, and tetraploid) with equal numbers of data lines. The dataset was partitioned into a training set comprising 70% of the data and a testing set comprising the remaining 30%. This partitioning facilitated model training on a subset of the data while allowing for independent validation of unseen data. Two input variables were considered for the ML models: oryzalin concentrations and exposure time. The output of the ML models consisted of three classes of polyploidy: diploid, mixoploid, and tetraploid. These classes were determined based on the observed polyploidy levels in the experimental instances.

### 4.4. Machine Learning Algorithms

Three classification-based machine learning algorithms—probabilistic neural network (PNN), support vector classification (SVC), and k-nearest neighbors (KNN)—were used to predict ploidy levels.

#### 4.4.1. Probabilistic Neural Network (PNN)

The probabilistic neural network (PNN) is a type of artificial neural network particularly suited for pattern classification tasks. It comprises four layers: input, pattern, summation, and output (Figure 5C).

The PNN architecture can be described by the following equations:

Input Layer: The input layer receives the input variables, $x_{1}$ and $x_{2}$, corresponding to oryzalin concentrations and exposure time, respectively.

Pattern Layer: The pattern layer computes the Euclidean distance between the input vector xxx and each training sample $x_{i}$ in the training set, i=1, 2, 3, …, n. This layer is represented by the equation:

$$f\left(x,x_{i}\right)=\exp\;(\frac{-\|x-x_{i}{\|}^{2}}{2\sigma^{2}})$$

where $\|x-x_{i}{\|}^{2}$ denotes the squared Euclidean distance between the input vector x and the training sample $x_{i}$, and σ is a smoothing parameter.

Summation Layer: The summation layer sums the outputs of the pattern layer for each class, weighted by the corresponding class probabilities. This layer is represented by the equation:

$$S_{j}=\overset{N_{j}}{\underset{i=1}{\sum }}f\left(x,x_{i}\right)$$

where $S_{j}$ is the summed activation for class j, $N_{j}$ is the number of training samples in class j, and $f\left(x,x_{i}\right)$ is the output of the pattern layer for the $i^{th}\;$ training sample in class $f\left(x,x_{i}\right)$.

Output Layer: The output layer computes the posterior probability of each class given the input vector xxx. This layer is represented by the equation:

$$P\;\left(C_{j}|x\right)=\frac{S_{j}}{\sum_{k=1}^{K}S_{k}}$$

where $P\;\left(C_{j}|x\right)$ is the posterior probability of class j given the input vector x, $S_{j}$ is the summed activation for class j, and K is the total number of classes.

The PNN model was trained using the training set, where the input variables and corresponding class labels were used to adjust the network parameters. Subsequently, the model was evaluated using the testing set to assess its performance in classifying polyploidy levels.

#### 4.4.2. Support Vector Classification (SVC)

Support vector classification (SVC) is a supervised learning algorithm used for classification tasks. It aims to find the hyperplane that best separates instances of different classes in a high-dimensional space (Figure 5D). The Radial Basis Function (RBF) kernel is commonly used in SVC to map input data into a higher-dimensional space, allowing for nonlinear decision boundaries.

The decision function for the SVC model with the RBF kernel can be represented as follows:

$$f\left(x\right)=sign\;(\sum_{i=1}^{n_{SV}}a_{i}y_{i}K\left(x_{i},\;x\right)+b)$$

where $f\left(x\right)$ is the decision function, $a_{i}$ are the coefficients determined during training, $y_{i}$ are the class labels, b is the bias term, $n_{SV}$ is the number of support vectors, and is the RBF kernel function, given by:

$$K\left(x_{i},\;x\right)=\exp\;{\left(-\gamma \|x-x_{i}\|\right)}^{2}$$

where γ is the kernel coefficient.

The SVC model with the RBF kernel was trained using the training set. During training, the algorithm identified the optimal hyperplane that separates the data into the three polyploidy classes based on the input variables: oryzalin concentrations and exposure time. Following training, the performance of the trained SVC model was evaluated using the testing set. The input variables from the testing set were fed into the trained model, and the predicted polyploidy classes were compared against the actual class labels to assess the model’s accuracy and generalization ability.

#### 4.4.3. K-Nearest Neighbors (KNNs)

K-nearest neighbors (KNNs) is a non-parametric supervised learning algorithm used for classification tasks. It operates by classifying an instance based on the majority class among its k-nearest neighbors in the feature space (Figure 5E). In this study, cosine distance was employed to calculate the similarity between instances, allowing for robust classification in high-dimensional spaces. The cosine distance between two vectors u and v is given by the following equation:

$$cosine_{distance}\left(u,\;v\right)=\frac{u\;.\;v}{\left\|u\|\;.\;\|v\right\|}$$

where u . v denotes the dot product and ‖u‖ and ‖v‖ are the magnitudes of vectors u and v, respectively.

The KNN model with cosine distance was trained using the training set. During training, the algorithm calculated the cosine distance between each instance in the training set and all other instances in the feature space. Following training, the performance of the trained KNN model was evaluated using the testing set. For each instance in the testing set, the algorithm identified its k-nearest neighbors from the training set using cosine distance and assigned the majority class label among those neighbors as the predicted class label.

### 4.5. Model Performance

The following performance criteria were used for evaluating and comparing the performance of the developed KNN, SVC, and PNN models:

$$Precision=\frac{TP}{TP+FP}$$

$$Accuracy=\frac{TP+TN}{TP+TN+FP+FN}$$

$$Recall=\frac{TP}{TP+FN}$$

$$Erro\;rate=\frac{FP+FN}{TP+TN+FP+FN}$$

$$F_{1}-Score=\frac{2\times Precision\times Recall}{Precision+Recall}$$

where *FN* is false negative, *FP* is false positive, *TN* is true negative, and *TP* is true positive.

### 4.6. Genetic Optimization Algorithm

In this study, the developed PNN model was utilized in conjunction with a genetic algorithm (GA) to determine the optimal levels of oryzalin and exposure time to produce tetraploid plants. The PNN model served as the fitness function for the GA, guiding the optimization process towards configurations that yield desired polyploidy outcomes.

The GA employed in this study was configured with specific parameters to govern its operation. These parameters were set as follows: (i) the initial population size was set to 200 individuals, representing candidate solutions, (ii) the GA iterated through 1000 generations (generation number) to evolve and refine the population towards optimal solutions, (iii) a mutation rate of 0.05 was applied to introduce variability in the population, promoting diversity and exploration of the solution space, (iv) the crossover rate, set at 0.6, determined the likelihood of crossover events between individuals during reproduction, (v) the creation function initialized the initial population uniformly within specified bounds, (vi) the crossover function implemented scattered crossover, allowing for diverse recombination of genetic information between individuals, (vii) the selection function employed stochastic uniform selection to probabilistically select individuals for reproduction, favoring individuals with higher fitness, and (viii) the mutation function adapted feasible mutation, ensuring that mutated individuals remained within predefined feasible bounds (Figure 5F).

MATLAB software (version R2023a) was employed to develop ML models and GA.

### 4.7. Validation Experiment

The predicted-optimized result obtained by PNN-GA was subjected to laboratory testing to assess the reliability and accuracy of the ML-assisted tetraploid induction in cannabis. The validation experiment comprised 4 replicates, each consisting of 4 explants. To do this, explants were first cultured in liquid DKW medium with 32.98 µM oryzalin for around 18 h (predicted-optimized condition). Subsequently, the treated explants were transferred to a solid DKW medium and kept in the growth room for 6 weeks. The leaves of the micro-propagated plants were then used for ploidy assessment using flow cytometry.

## 5. Conclusions

Artificial polyploid induction is a complex biological process influenced by various factors, including genotype, type and age of explants, type and concentration of antimitotic agents such as oryzalin, and exposure duration. Particularly, selecting the optimal oryzalin concentration and exposure time is the main prerequisite for successful polyploidization. Achieving a high rate of tetraploid induction with minimal mixoploid formation necessitates testing different combinations of oryzalin concentrations and exposure times through a factorial experiment that would be a tedious, cost- and time-consuming experiment; additionally, there is a risk that the optimal combination might lie outside the tested treatments. Data-driven approaches using ML can provide a powerful and reliable solution to this challenge. The results of the current study indicated that PNN outperformed both KNNs and SVC in predicting ploidy levels. Additionally, the hybrid PNN-GA accurately optimized oryzalin concentration and exposure duration to maximize tetraploid induction in the drug-type cannabis cultivar ‘Super Sherb’. Future studies should evaluate the suitability of this model and the optimized conditions for tetraploid induction in other commercial fiber-type and drug-type cannabis varieties.

## Figures and Tables

**Table 1:** Evaluation of k-nearest neighbors (KNNs), probabilistic neural network (PNN), and support vector classification (SVC) for predicting the level of ploidy in cannabis.

Performance Criteria	Training Set			Testing Set		
	KNN	PNN	SVC	KNN	PNN	SVC
Accuracy	92.9825%	96.4912%	80.7018%	80%	86.6667%	80%
Error rate	7.0175%	3.5088%	19.2982%	20%	13.3333%	20%
Precision	0.91238	0.95238	0.91273	0.8	0.93254	0.89599
Recall	0.87238	0.95238	0.74074	0.8	0.89725	0.57143
F_1_ Score	0.89193	0.95238	0.81779	0.8	0.91514	0.69782

## Data Availability

All relevant data are included in the paper.
